# Plasma p-tau217 as a biomarker of Alzheimer’s disease pathology in individuals with Down syndrome

**DOI:** 10.1038/s41467-025-65882-x

**Published:** 2025-11-10

**Authors:** Hanna Huber, Javier Arranz, Burak Arslan, Antoine Leuzy, Oscar Kittel, Guglielmo di Molfetta, Bessy Benejam, Laura Videla, Isabel Barroeta, Laura del Hoyo Soriano, Lucía Maure-Blesa, Íñigo Rodríguez-Baz, José Enrique Arriola Infante, Ignacio Illán-Gala, Alexandre Bejanin, Laia Montoliu-Gaya, Alberto Lleó, María Carmona-Iragui, Daniel Alcolea, Kaj Blennow, Henrik Zetterberg, Juan Fortea, Nicholas J. Ashton

**Affiliations:** 1 Institute of Neuroscience and Physiology, University of Gothenburg, Mölndal, Sweden; 2 German Center of Neurodegenerative Diseases (DZNE), Bonn, Germany; 3 Sant Pau Memory Unit, Department of Neurology, Hospital de la Santa Creu i Sant Pau, Biomedical Research Institute Sant Pau (IIB Sant Pau), Universitat Autònoma de Barcelona, Barcelona, Spain; 4 Banner Alzheimer’s Institute, Phoenix, AZ USA; 5 Banner Sun Health Research Institute, Sun City, AZ USA; 6 Barcelona Down Medical Center, Fundació Catalana Síndrome de Down, Barcelona, Spain; 7 Center of Biomedical Investigation Network for Neurodegenerative Diseases (CIBERNED), Madrid, Spain; 8 Department of Neurology, Torrecárdenas University Hospital, Almería, Spain; 9 Clinical Neurochemistry Laboratory, Sahlgrenska University Hospital, Mölndal, Sweden; 10 Paris Brain Institute, ICM, Pitié-Salpêtrière Hospital, Sorbonne University, Paris, France; 11 Division of Life Sciences and Medicine, and Department of Neurology, Institute on Aging and Brain Disorders, Neurodegenerative Disorder Research Center, University of Science and Technology of China and First Affiliated Hospital of USTC, Hefei, China; 12 Department of Neurodegenerative Disease, UCL Institute of Neurology, Queen Square, London, UK; 13 UK Dementia Research Institute at UCL, London, UK; 14 Hong Kong Center for Neurodegenerative Diseases, InnoHK, Hong Kong, China; 15 Wisconsin Alzheimer’s Disease Research Center, University of Wisconsin School of Medicine and Public Health, University of Wisconsin-Madison, Madison, WI USA

**Keywords:** Diagnostic markers, Alzheimer's disease, Alzheimer's disease

## Abstract

Diagnosing Alzheimer’s disease (AD) in adults with Down syndrome (DS), a population with a high genetically determined risk of AD, remains challenging. In this large observational study including *n* = 2329 samples from the Down Alzheimer Barcelona Neuroimaging Initiative (DABNI) and euploid controls from the Sant Pau Initiative on Neurodegeneration (SPIN) with and without symptomatic AD, we investigate if the strong diagnostic performance of plasma p-tau217 observed in sporadic AD extends to the DS population. Plasma p-tau217 discriminated cognitively stable individuals with DS from those with AD dementia with an AUC of 0.96 (95% CI, 0.95-0.97), and from those with prodromal AD with an AUC of 0.90 (95% CI, 0.87-0.92). Amyloid β (Aβ) positive and Aβ negative individuals with DS were distinguished with an AUC of 0.95 (95% CI, 0.92-0.99). In this study, we demonstrate that plasma p-tau217 is highly accurate in detecting amyloid β positivity and predicting clinical progression in individuals with DS, outperforming other plasma biomarkers. These findings support its use as a reliable, noninvasive tool for early AD detection and management in individuals with DS.

## Abstract (web summary)

The authors show that phosphorylated tau 217 is an accurate blood-based biomarker to detect Alzheimer’s disease and predict disease progression in individuals with Down syndrome, supporting its use as a reliable, non-invasive tool for early diagnosis and care.

## Introduction

Down syndrome (DS) is the most frequent cause of intellectual disability of genetic origin affecting 5.8 million people worldwide. Due to improved treatment of cardiac and hematological disease in DS, life expectancy has increased, and the leading cause of death in DS individuals nowadays is dementia^1–3^. It has been estimated that the lifetime risk of developing Alzheimer-type dementia in adults with DS is >90%^4,5^. The triplication of the amyloid precursor protein (*APP)* gene, located on chromosome 21, is sufficient to cause early-onset Alzheimer’s disease (AD), making DS the most prevalent genetically determined form of the condition^6^. By age 40, nearly all individuals with DS develop the core pathological hallmarks of AD, amyloid β (Aβ) plaques and neurofibrillary tau tangles^7^. Despite minor differences as the timing of initial amyloid accumulation, changes in amyloid positron emission tomography (PET) in DS appear to be comparable to autosomal dominant AD, which strongly supports early amyloid dysregulation in individuals with DS^8^. Diagnosing dementia in adults with DS is challenging, as dementia-associated cognitive decline is often obscured by the variability in intellectual disability^9^. Cerebrospinal fluid (CSF), molecular imaging, and now, blood biomarkers, have shown high diagnostic accuracy for detecting AD in DS while showing similar changes as in autosomal dominant AD^5,10–16^. However, despite these similarities in core AD biomarkers, DS is associated with a high number of comorbidities and complex medical conditions differentially affecting biochemical and hematological parameters compared to euploid individuals^17^. The most pressing clinical need in DS is the development of therapies to prevent or delay the onset of AD. Despite being an optimal population for prevention trials—due to their higher prevalence of AD compared to families with autosomal dominant AD and their more uniform pathophysiology than sporadic AD^1^—few trials have been conducted in individuals with DS.

Plasma phosphorylated tau217 (p-tau217) has recently emerged as a highly accessible and specific biomarker for the detection of biological AD, demonstrating concordance with CSF biomarkers and utility for monitoring longitudinal changes, including during the preclinical stage^18^. In this present observational study, we examine the diagnostic accuracy of p-tau217, and further blood biomarkers including p-tau231, p-tau181, Neurofilament light (NfL) and glial fibrillary acidic protein (GFAP), in detecting AD in DS.

Here, we show that plasma p-tau217 is a superior blood-based biomarker with high accuracy for the detection of Aβ pathology and the diagnosis of symptomatic AD in individuals with DS. Our findings highlight the potential of plasma p-tau217 for the early detection and monitoring of Aβ pathology and disease progression in individuals with DS, supporting its use in the recruitment of DS participants for therapeutic trials.

## Results

### Participant characteristics

Participant characteristics are summarized in Table 1 and Supplementary Table S1. A total of 2329 samples from 1372 participants were included in the study. This included 1332 samples from participants with DS (mean [SD] age at baseline, 42.7 [11.0] years; n [%] 614 females [46.1%], Table 1); 837 individuals were classified as asymptomatic (aDS), 145 as prodromal AD (pDS) and 351 as AD dementia (dDS); and 997 samples from euploid individuals (69.1 [11.1] years; n [%] 608 females [60.1%]) classified as either cognitively normal (CN; 350 individuals), mild cognitive impairment due to AD pathology (MCI-AD; 304 individuals) or AD dementia (343 individuals) at the respective visit. Participants attended mean [SD] 3.0 [1.2] visits (range, 2–8 visits) within mean [SD] 3.0 [2.3] years (range, 0–11 years).

**Table 1:** Baseline characteristics

	All	Individuals with Down syndrome				Euploid individuals			
		aDS	pDS	dDS	p-value^a^	CN	MCI-AD	AD	p-value^a^
Age, mean [SD], years	54.0 [16]	38.8 [9.5]	51.3 [5.2]	53.6 [5.4]	1.32e-60	57.0 [12.5]	73.2 [6.1]	72.8 [7.0]	6.48e-99
Sex, count, female /male	1222/1107	353/483	74/71	187/164	0.08	218/132	186/118	204/139	0.28
MMSE score, mean [SD]	24 [7]	NA	NA	NA	NA	29 [3]	25 [4]	20 [8]	7.84e-48
CSF Aβ42/40, mean [SD]	0.06 [0.03]	0.08 [0.02]	0.05 [0.01]	0.05 [0.01]	3.58e-25	0.10 [0.02]	0.04 [0.01]	0.05 [0.01]	1.14e-190
Plasma p-tau217, mean [SD], pg/mL	1.12 [1.14]	0.56 [0.48]	1.79 [0.99]	2.89 [1.47]	1.48e-90	0.33 [0.16]	0.99 [0.48]	1.27 [0.72]	4.32e-51
Plasma p-tau231, mean [SD], pg/mL	13.5 [9.4]	10.1 [5.7]	16.8 [7.3]	22.6 [10.7]	4.39e-43	9.3 [8.9]	14.5 [7.4]	17.3 [10.2]	0.06
Plasma p-tau181, mean [SD], pg/mL	18.1 [12.9]	13.0 [10.4]	23.1 [10.6]	31.0 [12.6]	5.15e-48	12.3 [9.9]	21.8 [11.4]	22.6 [9.9]	2.23e-07
Plasma NfL, mean [SD], pg/mL	17.0 [15.8]	11.6 [7.6]	21.7 [10.8]	34.2 [20.3]	8.36e-50	10.4 [8.2]	15.8 [7.6]	23.0 [25.2]	9.75e-14
Plasma GFAP, mean [SD], pg/mL	109.1 [72.1]	109.9 [73.0]	224.7 [128.3]	368.8 [208.1]	4.41e-60	110.8 [54.1]	218.0 [93.0]	268.6 [114.0]	2.55e-27
Centiloids	42.1 [42.6]	23.5 [32.7]	75.4 [37.3]^a^	71.1 [36.9]^a^	1.82e-05	23.0 [51.2]	56.5 [25.1]	73.6 [47.7]	0.45
FU time, median [SD], years	3.0 [1.2]	3.5 [2.3]	3.0 [1.8]	2.0 [1.9]	0.01	4.0 [2.2]	2.0 [0.5]	2.0 [0.5]	0.03
Number of visits, median [SD], years	3.0 [2.3]	3.0 [1.2]	3.0 [1.5]	3.0 [1.4]	3.97	3.0 [1.0]	2.0 [2.0]	2.0 [2.4]	1.69e-03

P-values have been corrected for multiple testing using Bonferroni correction.

In the DS cohort, no MMSE was performed; imaging biomarkers were available for a subgroup (*n* = 102).

*Aβ* amyloid beta, *AD* Alzheimer’s disease *aDS* asymptomatic Down syndrome, *CN* cognitively normal, *CSF* cerebrospinal fluid, *dDS* Down syndrome with dementia, *f* female, *FU* follow-up, *GFAP* glial fibrillary acidic protein, *MCI* mild cognitive impairment, *m* male, *MMSE* minimental state examination, *NfL* neurofilament light, *pDS* presymptomatic Down syndrome, *p-tau* phosphorylated tau.

^a^P-value refers to the comparison of the 3 diagnostic groups, results from 1-way ANOVA.

^b^Compared using Pearson’s Chi Square test.

^a^no significant difference was found for pDS (*n* = 10) vs. dDS (*n* = 13) group comparison from two-sided t-test (P = 0.7891; t = 0.2709, df = 21).

### Plasma p-tau217 levels by clinical diagnosis and accuracy in discriminating diagnostic groups

**Fig. 1:**
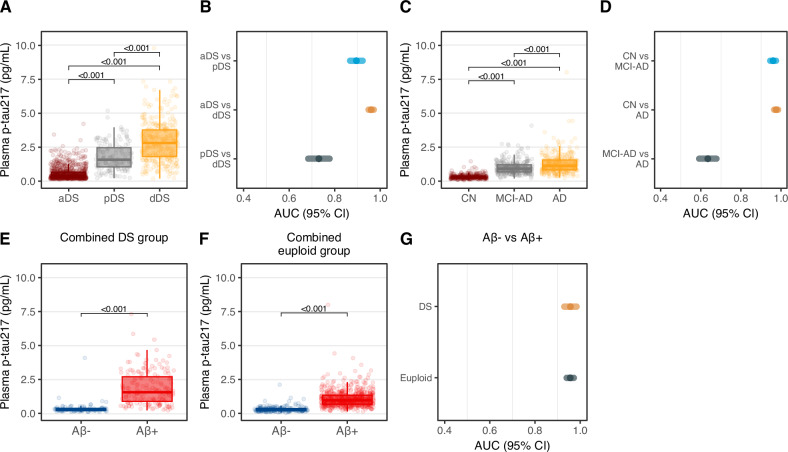
Plasma levels and diagnostic accuracy of p-tau217 in individuals with DS and euploid controls with and without AD pathology. Presented are the plasma concentrations (**A**, **C**) and the diagnostic performance (**B**, **D**) of p-tau217 in the clinical groups in individuals with Down syndrome and euploid controls. Show the plasma concentrations of p-tau217 in Aβ positive and negative individuals with (**E**) and without DS (**F**). In **G** the diagnostic accuracy of plasma p-tau217 in discriminating Aβ positive and negative individuals in both cohorts is presented. In **A**, **C** the cognitively stable individuals are presented in burgundy, mildly cognitively impaired individuals in gray and individuals with dementia in yellow. In **B**, **D**, **G** the cognitively stable group is plotted in cyan, the mildly cognitively impaired group in light peach and the dementia group in gray. In **E**, **F** the Aβ-group is displayed in blue, the Aβ+ group is displayed in red. aDS vs. dDS, *n* = 1154; aDS vs. pDS, *n* = 955; pDS vs. dDS, *n* = 483; Aβ+ vs. Aβ, *n* = 266. Exact p-values: **A**: aDS vs pDS, p = 2.09E-30; aDS vs dDS, p = 5.36E-96; pDS vs dDS, p = 2.29E-19; **C**: CN vs MCI, p = 6.18E-73; CN vs AD, p = 2.62E-73; MCI vs AD, p = 2.44E-08; **E**: p = 3.06E-34, **F**: p = 2.59E-114. P-values were derived from two-sided independent t-tests for pairwise group comparisons; p-values were not corrected for multiple testing. Boxplots display the median, IQR (bounds of the box), and whiskers extending to the minimum and maximum values within 1.5 × IQR; individual data points are shown with jittered dots (**A**, **C**, **E**, **F**). The performance of plasma biomarkers in predicting the diagnostic and Aβ groups was analyzed using AUC ROC analyses; the AUC and respective 95% CI are presented in the forest plot (**B**, **D**, **G**). Source data are provided as a Source data file. Aβ amyloid beta, AD Alzheimer’s disease, aDS asymptomatic Down syndrome, AUC area under the curve, CI confidence interval, CN cognitively normal, CSF cerebrospinal fluid, dDS Down syndrome with dementia, IQR interquartile range, MCI mild cognitive impairment, pDS presymptomatic Down syndrome, ROC receiver operating characteristics.

In participants with DS, the highest plasma p-tau217 concentration was observed in dDS (mean [SD], pg/mL, 2.87 [1.42]) and pDS (1.79 [0.99]) which could be distinguished from aDS (0.56 [0.48]) with high accuracy (AUC 0.96 [95% CI, 0.95–0.97] and 0.90 [95% CI, 0.87–0.92], respectively, Fig. 1 and Table 2). Plasma p-tau217 levels across age in individuals with DS are presented in Supplementary Fig. S1. In the euploid individuals, we found a similar pattern with the highest p-tau217 levels in AD (mean [SD], pg/mL, 1.25 [0.62]), followed by MCI-AD (0.99 [0.48]) and CN (0.33 [0.16]). The levels were substantially lower compared to DS. The discrimination of cognitively unimpaired and prodromal/demented individuals (CN vs. AD, AUC, 0.97; 95% CI, 0.96–0.98; CN vs. MCI-AD, AUC, 0.96; 95% CI, 0.95–0.97) was highly accurate. The AUCs of prodromal vs. symptomatic groups are presented in Table 2. Next, we derived a binary p-tau217 cut-off for the clinical diagnosis (aDS vs. dDS) in the DS cohort using the Youden index (>1.46 pg/mL; Youden index, 0.79; sensitivity, 84.8%; specificity, 94.1%, NPV, 85.1%, PPV, 84.8%). In the euploid cohort, the cut-off value (CN vs. AD) was >0.61 pg/mL (Youden index, 0.85; sensitivity, 89.2%; specificity, Table 2).

**Table 2:** Discriminative performance and cut-off values of p-tau217 in DS and euploid individuals with and without AD pathology

	AUC [95% CI]	Participant samples (N)	Cut-off p-tau217 level (pg/mL)	Youden index	Sensitivity [95% CI] (%)	Specificity [95% CI] (%)	Likelihood ratio
Individuals with Down syndrome							
Clinical diagnosis							
aDS vs. dDS	0.96 [0.95–0.97]	1154	1.460	0.789	84.8 [80.6–88.2]	94.1 [92.3–95.5%]	14.35
aDS v. pDS	0.90 [0.87–0.92]	955	0.695	0.657	89.4 [83.3–93.5]	76.36 [73.2–79.1]	3.77
pDS vs. dDS	0.76 [0.71–0.80]	483	1.775	0.351	76.0 [71.1–80.2]	59.2 [50.9–66.9]	1.86
Aβ status							
Aβ+ vs. Aβ	0.95 [0.92–0.98]	266	0.585	0.791	87.0 [81.3–91.2]	92.1 [84.6–96.1]	11.06
Euploid individuals							
Clinical diagnosis							
CN vs. AD	0.97 [0.96–0.98]	660	0.605	0.846	89.2 [85.4–92.1]	95.4 [92.6–97.2]	19.56
CN v. MCI-AD	0.96 [0.95–0.97]	632	0.495	0.796	91.4 [87.7–94.1]	88.2 [84.21–91.2]	7.71
MCI-AD vs. AD	0.63 [0.59–0.68]	634	1.015	0.219	59.2 [53.8–64.4]	62.7 [57.1–68.0]	1.59
Aβ status							
Aβ+ vs. Aβ	0.96 [0.94–0.97]	821	0.495	0.811	89.3 [84.5–92.8]	91.8 [89.3–93.7]	10.82

AUC from ROC analyses discriminating clinical groups and CSF/PET amyloid positive/negative individuals. AUC and 95% CI are presented. Youden’s J statistics were used for cut-off points selection

*Aβ* amyloid beta, *AD* Alzheimer’s disease, *aDS* asymptomatic Down syndrome. *AUC* area under the curve, *CI* confidence interval, *CN* cognitively normal, *CSF* cerebrospinal fluid, *dDS* Down syndrome with dementia, *MCI* mild cognitive impairment, *pDS* presymptomatic Down syndrome, *ROC* receiver operating characteristics.

### Plasma p-tau217 levels by amyloid status and accuracy in discriminating abnormal Aβ

For a subset of 1061 samples (*n* = 772 euploid individuals, *n* = 289 DS individuals) paired CSF (*n* = 959) and/or amyloid PET biomarkers (*n* = 102) were available. In DS participants, plasma p-tau217 was 4.7-fold higher in Aβ+ vs. Aβ− individuals (mean [SD], pg/mL, 1.86 [1.23] vs. 0.39 [0.44]) and predicted abnormal CSF Aβ42/40 or amyloid PET with high accuracy (Aβ+ vs. Aβ+, AUC, 0.95; 95% CI, 0.92–0.99). Notably, p-tau217 levels of Aβ− asymptomatic individuals where similar in the DS and euploid groups (mean [SD], pg/mL, 0.33 [0.14] in DS; 0.31 [0.13] in the euploid cohort), while p-tau217 levels in Aβ+ asymptomatic individuals where substantially higher in DS (mean [SD], pg/mL, 0.88 [0.53]) compared to the euploid group (mean [SD], pg/mL, 0.49 [0.29]). Once more, euploid individuals followed the same pattern with plasma p-tau217 levels being 3.4-fold higher in Aβ+ compared to Aβ− individuals (mean [SD], pg/mL, 1.10 [0.62] vs. 0.33 [0.20]) and an AUC of 0.96 (95% CI, 0.94–0.97, Fig. 1 and Table 2). The optimal cut-off for defining Aβ positivity using p-tau217 was >0.59 pg/mL in DS (Youden index 0.79; sensitivity, 87.0%; specificity, 92.1%) and >0.50 pg/mL in the euploid cohort (Youden index 0.81; sensitivity, 89.3%; specificity, 91.8%). In line with current literature^19,20^, analyses adjusted for age and sex did not substantially differ from unadjusted results; adjusted data as well as predictive value of age alone in the DS group are reported in Supplementary Table S2. Discriminative performance of plasma p-tau217 in individuals with DS was consistently high when using different threshold for amyloid PET positivity (AUC [95% CI]; 18 CL: 0.92 [0.82–1]; 25 CL: 0.96 [0.88–1]; 30 CL: 0.96 [0.89–1]; 35 CL: 0.95 [0.87–1]).

### Plasma p-tau217 compared to p-tau181, p-tau231, NfL and GFAP

Plasma p-tau181, p-tau231, NfL, and GFAP levels increased progressively based on clinical diagnosis, with the lowest concentrations observed in aDS, and the highest concentrations observed in dDS. This pattern closely mirrored findings in the euploid cohort (Supplementary Figs. S1–S4).

GFAP demonstrated the highest AUC among p-tau181, p-tau231 and NfL in predicting clinical outcomes (aDS vs. dDS; AUC, 0.95; 95% CI, 0.93–0.96) and identifying abnormal Aβ status (AUC, 0.88; 95% CI, 0.83–0.93) within the DS cohort (Supplementary Figs. S1–S4). Among these biomarkers in the DS cohort, p-tau231 presented the weakest accuracy in predicting clinical stage (aDS vs. dDS; AUC, 0.87; 95% CI, 0.85–0.90) and Aβ positivity (AUC, 0.79; 95% CI, 0.74–0.85). Comparable biomarker performance was also observed in the euploid cohort. Compared to p-tau217, GFAP, p-tau181, p-tau231, and NfL showed significantly lower accuracy in predicting disease progression in DS and euploid individuals (aDS vs. dDS; p-tau217 vs. p-tau181, mean difference between areas [95% CI], p-value from DeLong comparison, 0.05 [0.03–0.06], p < 0.0001; p-tau217 vs. p-tau231, 0.09 [0.06–0.11], p < 0.0001; p-tau217 vs. NfL, 0.03 [0.02–0.05], p < 0.0001, p-tau217 vs. GFAP, 0.02 [0.00–0.03], p = 0.014; and CN vs. AD; p-tau217 vs. p-tau181, mean difference between areas [95% CI], p-value from DeLong comparison, 0.12 [0.08–0.15], p < 0.0001; p-tau217 vs. p-tau231, 0.14 [0.09–0.18], p < 0.0001; p-tau217 vs. NfL, 0.11 [0.08–0.15], p < 0.0001, p-tau217 vs. GFAP, 0.08 [0.04–0.11], p < 0.0001; Supplementary Figs. S2–S5; Supplementary Tables S3 and S4). Accordingly, p-tau217 outperformed the other plasma biomarkers in predicting Aβ positivity (DS Aβ+ vs Aβ−, p-tau217 vs. p-tau181, mean difference between areas [95% CI], p-value from DeLong comparison, 0.13 [0.08–0.17], p < 0.0001; p-tau217 vs. p-tau231, 0.13 [0.08–0.18], p < 0.0001; p-tau217 vs. NfL, 0.13 [0.06–0.16], p < 0.0001, p-tau217 vs. GFAP, 0.09 [0.04–0.14], p = 0.0003; euploid Aβ+ vs Aβ− for p-tau217 vs. p-tau181, mean difference between areas [95% CI], p-value from DeLong comparison, 0.12 [0.07–0.17], p < 0.0001; p-tau217 vs. p-tau231, 0.13 [0.05–0.18], p < 0.0001; p-tau217 vs. NfL, 0.09 [0.05–0.14], p = 0.0001, p-tau217 vs. GFAP, 0.07 [0.03–0.11], p = 0.0012; Supplementary Figs. S2–S5; Supplementary Tables S3 and S4).

Combining plasma biomarkers (all biomarkers, p-tau217 + NfL + GFAP, p-tau217 +  NfL, and p-tau217 + GFAP) did not substantially increase diagnostic accuracy for clinical stage and amyloid positivity (aDS vs. dDS, p-tau217 alone AUC [95% CI], 0.96 [0.95–0.97], p-tau217 + GFAP + NfL + p-tau231 + p-tau181, AUC [95% CI], 0.97 [0.96–0.98]; DS Aβ+ vs. Aβ−, p-tau217 alone, AUC [95% CI], 0.95 [0.92–0.98], p-tau217 + GFAP + NfL + p-tau231 + p-tau181, AUC [95% CI], 0.95 [0.92–0.98]; Supplementary Table S5).

## Discussion

Plasma p-tau217 showed similarly high accuracy in detecting Aβ pathology and disease progression in individuals with DS compared to those in a euploid population, though the optimal cut-off thresholds were higher in DS. Furthermore, plasma p-tau217 outperformed the other tested plasma biomarkers in predictive accuracy. Our findings highlight the potential of plasma p-tau217 for the early detection and monitoring of Aβ pathology and symptomatic AD in individuals with DS, supporting its use in the recruitment of DS participants for therapeutic trials.

Recent approvals of anti-amyloid monoclonal antibodies for AD are backed by strong evidence from randomized, placebo-controlled trials demonstrating that reducing Aβ plaque load can lead to improved outcomes for individuals with early-stage AD^21,22^. Despite this progress, individuals with DS and AD-type pathology have been excluded from these trials^23^. There is now momentum for including the DS population in disease-modifying or prevention trials^23^, similar to trials with *PSEN1* or *APP* mutation carriers. Biomarkers, especially minimally invasive blood biomarkers, will play a critical role in recruitment and may also help assess outcomes^10^.

This study emphasizes the strong diagnostic performance and potential usefulness of plasma p-tau217 in individuals with DS, aligning with findings in sporadic AD^18,24^. We demonstrated that p-tau217 had a similarly good performance in identifying true positive (Aβ+) and true negative (Aβ−) DS individuals (NPV/ PPV, 85.1%/ 84.8%, respectively). However, when assessing symptomatic AD diagnosis, NfL^15^, GFAP^11^ and p-tau212^25^ also showed high accuracy. In earlier studies with smaller cohorts, plasma p-tau217 was identified as superior biomarker of tau pathological brain changes^26^ and predicted Aβ accumulation and progression to dementia in DS^27^. Consistent with our findings that GFAP had the second highest predictive accuracy for clinical stage and amyloid status among the biomarkers we investigated, plasma GFAP was reported to be associated with Aβ accumulation in DS^27^.

Notably, as shown in this present study, the optimal biomarker cutoffs for AD pathology in DS differ; p-tau217 levels were substantially higher in the DS compared to the euploid cohort, which might be due to a higher amyloid and tau burden in DS^28,29^. Similarly, with a p-tau217 value of 0.59 pg/mL achieving 87.0% sensitivity and 92.1% specificity, a significantly higher threshold to detect Aβ abnormality was observed in DS compared to the euploid group (0.50 pg/mL; 89.3% sensitivity, 91.8% specificity). This was also true for the clinical diagnosis, possibly due to the presence of preclinical AD in the aDS group, as supported by substantially higher p-tau217 levels in asymptomatic Aβ+ individuals in the DS compared to the euploid group and the aforementioned higher amyloid and tau burden in DS^28,29^. The accuracy of p-tau217 in discriminating pDS from dDS group was, in line with other biomarker findings^25^, substantially lower than for other group comparisons—which may result from a slight underrepresentation of pDS individuals in our dataset, likely reflecting the rapid progression from pDS to dDS over only a few years.

In this study, the predictive accuracy of p-tau217 did not differ when adjusted for sex compared to unadjusted results. This is in line with earlier findings, where biological sex did not influence clinical and biomarker profiles of AD in adults with DS^20,30^. Despite the evidence of sex differences in disease onset/progression and various disease mechanisms during aging in sporadic AD^31–33^, its influence seems to be lesser in genetic forms of AD.

A limitation of this study is that these cutoff values for the ALZpath p-tau217 are only applicable for this specific test and cannot be translated to other immunoassays detecting p-tau217, e.g., the Lumipulse G pTau217 Plasma test. Moreover, not for all individuals CSF or, especially for this cohort, amyloid PET imaging biomarker confirmation was available, which is often a challenge in this population. Evaluation of longitudinal biomarker trajectories was not in the scope of this analysis.

To conclude, the results of this present study illustrate that plasma p-tau217 is an accurate and widely accessible blood-based biomarker with significant potential for the early detection and monitoring of Aβ pathology and disease progression in individuals with DS, supporting its use in the recruitment of DS participants for therapeutic trials.

## Methods

### Participants

Before starting the study, all protocols, participant information, and consent forms are approved by the Sant Pau Research Ethics Committee (IBSP-NGF-2018-36 and IIBSP-DOW-2014-30). Investigators provide detailed explanations of the study’s purpose, methods, and potential, and participants can request additional information or withdraw at any time. Before enrollment, consent and assent are obtained from the participants and their legally authorized representatives for collecting, analyzing, and storing biological samples, with participants informed about possible sharing of anonymized data and samples with other researchers. Confidentiality is maintained according to Spanish law (LOPD 3/2018). All study procedures comply with the principles of the Declaration of Helsinki^34^.

Participants with DS with or without symptomatic AD were recruited from a population-based healthcare program that involves annual neurological and neuropsychological evaluations. Adults with DS of both sexes over the age of 18 years^5,34^ who expressed interest in research were included in the Down Alzheimer Barcelona Neuroimaging Initiative (DABNI). Euploid controls with or without symptomatic AD were recruited from the Sant Pau Initiative on Neurodegeneration (SPIN)^35^ cohorts at the Sant Pau Memory Unit, Barcelona, Spain. Both cohorts are followed annually, and all data, including repeated measurements during follow-up visits, have been included in the present analysis to evaluate the performance of blood biomarkers in predicting clinical outcome. Euploid participants were classified as cognitively normal (CN), mild cognitive impairment due to AD pathology (MCI-AD) or AD dementia (AD); participants with DS were classified as asymptomatic (aDS), with prodromal AD (pDS) or with AD dementia (dDS) after an extensive medical history revision, a physical examination, and a neuropsychological assessment as described previously^5,15,34^. In DS individuals, the two preliminary diagnoses made by two blinded neurologists and neuropsychologists based on clinical and neuropsychological information were reviewed in a consensus clinical meeting and a unified diagnosis was established. Clinical classification and study procedures are performed at each visit. In a subgroup, CSF and imaging AD biomarkers are collected. Sex of participants was determined based on self-report. Participants have not been financially compensated for participating in the study.

### Plasma, CSF, and imaging biomarkers

Plasma biomarkers were quantified in singlecates using Single Molecule array (Simoa) technology; p-tau217 concentration was measured with the ALZpath p-tau217 v2 assay^18^ (104371; Quanterix, Billerica, MA, US); p-tau231 with an in-house assay developed at the Neurochemistry Laboratory, University Gothenburg, Sweden^36^; p-tau181 with the p-tau181 v2.1 assay (104111; Quanterix, Billerica, MA, US), and NfL and GFAP with the Neurology-2-Plex-B assay (103520; Quanterix, Billerica, MA, US) by scientists blinded to participant information. Plasma samples were diluted 4-fold for the Neurology-2-Plex B assay and 2-fold for all p-tau assays. For all biomarkers, the intra-assay CV was ≤ 6.1% and the inter-assay CV was ≤7.0%.

Aβ-positivity (Aβ+) was defined as CSF Aβ42/40 ratio <0.072 (*n* = 1028)^37^, CSF Aβ42 < 550 pg/mL (*n* = 77; Fujirebio’s Lumipulse G600II) or Centiloid > 30 on amyloid PET using [^18^F]-florbetapir (*n* = 102)^38^ irrespective of cognitive status. Discriminative performance of plasma p-tau217 in individuals with DS has been tested for four additional thresholds (18 CL, 25 CL, 35 CL).

### Statistics and reproducibility

Data are shown as mean and standard deviation unless otherwise described. Group differences were assessed using one-way ANOVA for continuous variables and Pearson’s Chi Square test for categorical variables. Pairwise comparisons were adjusted for multiple testing using the Bonferroni correction. The performance of plasma biomarkers in predicting the diagnostic and Aβ groups was evaluated using area under the curve (AUC) values receiver-operating characteristic (ROC) analyses and DeLong’s test to compare AUC values for different biomarkers. All available biomarker measurements were included and treated as independent observation to enhance sensitivity in diagnostic classification by maximizing data usage from repeated assessments. The evaluation of longitudinal trajectories of the blood biomarkers was therefore not in the scope of this analysis. No statistical method was used to predetermine sample size. No data were excluded from analysis. Analyses adjusted for age and sex using binary logistic regression models did not significantly differ from unadjusted results; adjusted data is reported in the Supplementary section. Exact sample sizes for each group comparison are reported in Table 2. Youden’s J statistic was used for cut-off point selection. Positive (PPV) and negative predictive value (NPV) for plasma p-tau217 positivity were assessed in relation to clinical diagnosis.

All statistical analyses were performed using GraphPad Prism 9 for Windows, MedCalc version 23.0.9, or R version 4.3.3. The following R packages have been used: ggplot2, dplyr, ggsignif, pROC, and ggsci. Significance levels were set at P < 0.05.

### Reporting summary

Further information on research design is available in the Nature Portfolio Reporting Summary linked to this article.

## Supplementary information

Supplementary Information

Reporting Summary

Transparent Peer Review File

## Source data

Source data

## Data Availability

Due to consenting issues and IRB requirements, we cannot make the dataset publicly available in a repository, as explicit consent for public sharing was only introduced recently in our cohort and is therefore not in place for many participants. However, the DABNI cohort has supported dozens of international collaborations, and data can be shared following standard data transfer agreements. Requests can be directed to Juan Fortea (jfortea@santpau.cat) or Ana Bueno (abueno@santpau.cat). Access is limited to bona fide researchers for scientific purposes, and requests are typically processed and accepted within 2–3 months. Biomarker data displayed in this study are provided in the Supplementary Information/Source data file. Source data are provided with this paper.
