# Supplementary material for: Plasma p-tau217 as a biomarker of Alzheimer’s disease pathology in individuals with Down syndrome
Source: Nat Commun. 2025 Nov 10;16:9900. doi: 10.1038/s41467-025-65882-x (PMC12603275; doi:10.1038/s41467-025-65882-x)
Supplement: Supplementary file 1 — Supplementary Information [file 41467_2025_65882_MOESM1_ESM.pdf]

Plasma p-tau217 as a Biomarker of Alzheimer's Disease Pathology in Individuals with Down Syndrome

Hanna Huber[1,2], Javier Arranz[3], Burak Arslan[1], Antoine Leuzy[1,4,5], Oscar Kittel[1], Guglielmo di Molfetta[1], Bessy Benejam[3,6], Laura Videla[3,6,7], Isabel Barroeta[3,7], Laura del Hoyo Soriano[3], Lucía Maure-Blesa[3], Íñigo Rodríguez-Baz[3], José Enrique Arriola Infante[7,8], Ignacio Illán-Gala[3,7], Alexandre Bejanin[3,7], Laia Montoliu-Gaya[1], Alberto Lleó[3,7], María Carmona-Iragui[3], Daniel Alcolea[3,7], Kaj Blennow[1,9,10,11], Henrik Zetterberg[1,9,12,13,14,15], Juan Fortea[3,6,7*], Nicholas J. Ashton[1,4,5*+]

* equally contributed as senior authors

AFFILIATIONS

[1] Institute of Neuroscience and Physiology, University of Gothenburg, Mölndal, Sweden; [2] German Center of Neurodegenerative Diseases, Bonn, Germany; [3] Sant Pau Memory Unit, Department of Neurology, Hospital de la Santa Creu i Sant Pau, Biomedical Research Institute Sant Pau (IIB Sant Pau), Universitat Autònoma de Barcelona, Spain; [4] Banner Alzheimer's Institute, Phoenix, AZ, USA; [5] Banner Sun Health Research Institute, Sun City, AZ, USA; [6] Barcelona Down Medical Center, Fundació Catalana Síndrome de Down, Barcelona, Spain; [7] Center of Biomedical Investigation Network for Neurodegenerative Diseases (CIBERNED), Madrid, Spain; [8] Department of Neurology, Torrecárdenas University Hospital, Almería, Spain; [9] Clinical Neurochemistry Laboratory, Sahlgrenska University Hospital, Mölndal, Sweden; [10] Paris Brain Institute, ICM, Pitié-Salpêtrière Hospital, Sorbonne University, Paris, France; [11] Neurodegenerative Disorder Research Center, Division of Life Sciences and Medicine, and Department of Neurology, Institute on Aging and Brain Disorders, University of Science and Technology of China and First Affiliated Hospital of USTC, Hefei, P.R. China; [12] Department of Neurodegenerative Disease, UCL Institute of Neurology, Queen Square, London, UK; [13] UK Dementia Research Institute at UCL, London, UK; [14] Hong Kong Center for Neurodegenerative Diseases, InnoHK, Hong Kong, China; [15] Wisconsin Alzheimer's Disease Research Center, University of Wisconsin School of Medicine and Public Health, University of Wisconsin-Madison, Madison, WI, USA; [*] These authors contributed equally as senior authors

[+]Corresponding author:

Nicholas J. Ashton, PhD

Email: nicholas.ashton@gu.se

Phone: 480-703-5889

SUPPLEMENTARY MATERIAL

Table of Contents

Page 1 ─ Supplementary Table S1. Participant characteristics at baseline and during follow-up in DS and euploid clinical groups

Page 2 ─ Supplementary Table S2. Discriminative performance of p-tau217 unadjusted and adjusted for age and sex and the discriminative performance of age alone in DS individuals with and without AD pathology.

Page 3 ─ Supplementary Table S3. Accuracy of blood biomarkers in predicting clinical classification groups and amyloid status in DS and euploid individuals.

Page 4 ─ Supplementary Table S4. Accuracy of plasma p-tau217 vs. other blood biomarkers in predicting clinical diagnostic groups and amyloid status in individuals with Down syndrome and euploid individuals.

Page 5 ─ Supplementary Table S5. Accuracy of combined blood biomarkers in predicting clinical classification groups and amyloid status in DS individuals.

Page 6 ─ Supplementary Figure S1. Plasma p-tau217 levels across age.

Page 7 ─ Supplementary Figure S2. Plasma levels and diagnostic accuracy of p-tau231 in individuals with DS and euploid controls with and without AD pathology.

Page 8 ─ Supplementary Figure S3. Plasma levels and diagnostic accuracy of p-tau181 in individuals with DS and euploid controls with and without AD pathology.

Page 9 ─ Supplementary Figure S4. Plasma levels and diagnostic accuracy of NfL in individuals with DS and euploid controls with and without AD pathology.

Page 10 ─ Supplementary Figure S5. Plasma levels and diagnostic accuracy of GFAP in individuals with DS and euploid controls with and without AD pathology.

Supplementary Table S1. Participant characteristics at baseline and during follow-up in DS and euploid clinical groups.

	Group	Visit 1	Visit 2	Visit 3	Visit 4	Visit 5	Visit 6	Visit 7	Visit 8
**Age, mean [SD], years**	aDS	38.0 [9.9]	39.1 [9.4]	39.7 [9.0]	40.6 [8.4]	38.9 [7.7]	40.3 [8.2]	40.3 [4.2]	NA
	pDS	50.6 [5.3]	51.0 [4.4]	52.8 [5.4]	52.1 [5.3]	53.1 [5.8]	55.5 [9.2]	NA	NA
	dDS	52.7 [5.7]	53.7 [5.3]	53.4 [5.5]	54.7 [4.4]	55.5 [4.2]	56.9 [3.6]	58.6 [5.7]	57.0 [9.9]
	CN	55.1 [12.5]	57.1 [12.4]	60.2 [12.4]	61.2 [12.2]	66.0 [6.9]	65 [NA]	NA	NA
	AD-MCI	73.3 [6.1]	71.4 [6.7]	NA	74.0 [NA]	NA	NA	NA	NA
	AD	72.9 [7.1]	71.9 [5.9]	72.0 [6.8]	NA	NA	NA	NA	NA
**Sex, count, N male/ female**	aDS	179/221	89/131	48/75	24/31	9/18	3/4	1/2	0/0
	pDS	34/31	20/18	10/9	6/8	3/4	1/1	0/0	0/0
	dDS	68/72	49/39	31/26	16/16	11/6	7/3	4/1	1/1
	CN	109/62	56/40	34/19	15/8	3/3	1/0	0/0	0/0
	AD-MCI	179/108	7/9	0/0	0/0	0/0	0/0	0/0	0/0
	AD	187/120	16/15	1/4	0/0	0/0	0/0	0/0	0/0
**CSF Aβ42/40, mean [SD]**	aDS	0.08 [0.02]	0.07 [0.02]	0.09 [0.02]	0.08 [0.02]	NA	NA	NA	NA
	pDS	0.05 [0.02]	NA	NA	NA	NA	0.04 [NA]	NA	NA
	dDS	0.05 [0.01]	0.05 [0.01]	0.04 [0.01]	0.05 [0.01]	0.04 [NA]	NA	NA	NA
	CN	0.10 [0.02]	0.10 [0.02]	0.10 [0.02]	0.10 [0.01]	0.11 [NA]	NA	NA	NA
	AD-MCI	0.04 [0.01]	0.04 [0.01]	NA	0.04 [NA]	NA	NA	NA	NA
	AD	0.05 [0.01]	0.05 [0.01]	0.05 [0.01]	NA	NA	NA	NA	NA
**Plasma p-tau217, mean [SD], pg/mL**	aDS	0.53 [0.47]	0.57 [0.49]	0.59 [0.48]	0.58 [0.43]	0.50 [0.40]	0.91 [0.81]	1.29 [0.94]	NA
	pDS	1.99 [1.11]	1.70 [0.92]	1.62 [0.81]	1.51 [0.94]	1.60 [0.80]	1.59 [0.76]	NA	NA
	dDS	2.76 [1.55]	2.94 [1.42]	2.96 [1.61]	2.90 [1.16]	3.29 [1.14]	3.26 [1.68]	2.67 [0.76]	1.62 [1.08]
	CN	0.32 [0.18]	0.34 [0.16]	0.33 [0.14]	0.31 [0.12]	0.26 [0.09]	0.34 [NA]	NA	NA
	AD-MCI	0.99 [0.49]	1.05 [0.32]	NA	1.45 [NA]	NA	NA	NA	NA
	AD	1.26 [0.73]	1.30 [0.75]	1.36 [0.28]	NA	NA	NA	NA	NA
**Plasma p-tau231 mean [SD], pg/mL**	aDS	10.3 [5.8]	10.1 [6.5]	10.0 [4.9]	9.3 [3.6]	9.3 [3.8]	11.9 [5.0]	10.0 [NA]	NA
	pDS	17.7 [7.3]	NA	NA	NA	NA	NA	NA	NA
	dDS	22.8 [12.0]	20.7 [9.3]	24.0 [9.8]	22.3 [7.1]	28.1 [14.3]	37.2 [1.9]	21.7 [6.7]	17.8 [NA]
	CN	10.9 [12.8]	7.9 [4.3]	8.3 [3.5]	8.8 [3.8]	8.7 [3.9]	10.1 [NA]	NA	NA
	AD-MCI	14.7 [7.8]	13.4 [5.0]	NA	12.5 [NA]	NA	NA	NA	NA
	AD	15.9 [6.9]	20.6 [15.6]	19.8 [12.8]	NA	NA	NA	NA	NA
**Plasma p-tau181 mean [SD], pg/mL**	aDS	12.6 [8.5]	13.1 [9.5]	14.6 [18.1]	12.7 [6.1]	11.3 [5.6]	14.7 [8.3]	22.6 [NA]	NA
	pDS	23.8 [11.1]	22.8 [9.7]	20.3 [11.0]	20.9 [11.1]	31.5 [3.2]	24.8 [4.4]	NA	NA
	dDS	28.6 [12.4]	31.6 [12.3]	33.8 [13.5]	30.8 [10.5]	39.3 [11.1]	49.7 [10.5]	33.1 [9.6]	27.3 [NA]
	CN	13.3 [11.4]	11.7 [10.2]	11.3 [5.0]	10.6 [3.9]	12.1 [8.6]	8.9 [NA]	NA	NA
	AD-MCI	21.7 [11.1]	22.4 [13.9]	NA	19.1 [NA]	NA	NA	NA	NA
	AD	21.9 [9.1]	23.5 [11.1]	30.0 [16.1]	NA	NA	NA	NA	NA
**Plasma NfL mean [SD], pg/mL**	aDS	11.3 [7.5]	11.8 [7.5]	12.1 [8.0]	13.1 [7.5]	10.8 [4.6]	18.3 [NA]	NA	NA
	pDS	22.0 [11.1]	22.3 [10.7]	21.5 [16.3]	17.7 [4.8]	22.9 [4.1]	23.3 [0.3]	NA	NA
	dDS	32.0 [19.0]	36.0 [16.9]	40.6 [30.8]	28.3 [12.2]	28.1 [11.9]	52.4 [NA]	24.3 [NA]	NA
	CN	10.9 [10.1]	9.5 [4.8]	10.2 [6.9]	11.0 [6.8]	9.4 [4.3]	7.4 [NA]	NA	NA
	AD-MCI	15.6 [7.4]	20.2 [10.1]	NA	9.4 [NA]	NA	NA	NA	NA
	AD	20.8 [13.1]	36.0 [59.6]	21.6 [10.0]	NA	NA	NA	NA	NA
**Plasma GFAP mean [SD], pg/mL**	aDS	109.9 [73.0]	107.6 [70.4]	112.2 [78.5]	106.1 [71.3]	99.8 [41.8]	104.3 [25.2]	76.9 [NA]	NA
	pDS	249.8 [128.2]	199.6 [114.0]	223.9 [156.9]	174.9 [123.5]	140.8 [42.7]	179.6 [NA]	NA	NA
	dDS	391.4 [233.5]	362.7 [205.3]	361.4 [191.0]	304.1 [146.2]	311.5 [110.4]	497.0 [140.7]	462.6 [151.6]	181.8 [NA]
	CN	99.4 [49.6]	107.3 [53.4]	137.1 [60.0]	126.3 [42.7]	143.3 [30.1]	115.5 [NA]	NA	NA
	AD-MCI	221.3 [96.9]	203.9 [74.8]	NA	175.9 [NA]	NA	NA	NA	NA
	AD	279.4 [117.5]	252.1 [108.0]	193.0 [44.2]	NA	NA	NA	NA	NA

Visit 1 refers to the baseline evaluation; visits 2 to 8 refer to the follow-up visits. Reported is the mean of all available data, also if not all visits have been completed yet. NA means that no participant has completed this particular visit yet or that no SD could be calculated as only one data point was available.

Aβ, amyloid beta; AD, Alzheimer's disease; aDS, asymptomatic Down syndrome; CN, cognitively normal; CSF, cerebrospinal fluid; dDS, Down syndrome with dementia; GFAP, glial fibrillary acidic protein; MCI, mild cognitive impairment; NA, not available; NfL, neurofilament light; SD, standard deviation; p-tau, phosphorylated tau

Supplementary Table S2. Discriminative performance of p-tau217 unadjusted and adjusted for age and sex and the discriminative performance of age alone in DS individuals with and without AD pathology.

	**p-tau217**		**age**		
	**AUC [95% CI] unadjusted**	**AUC [95% CI] adjusted for age and sex**	**AUC [95 % CI] age**	**AUC [95 % CI] age > 40 years**	**AUC [95 % CI] age > 50 years**
**Clinical diagnosis**					
aDS vs. dDS	0.97 [0.97–0.98]	0.97 [0.97–0.98]	0.92 [0.91-0.94]	0.86 [0.83-0.88]	0.72 [0.65-0.78]
aDS v. pDS	0.92 [0.90-0.94]	0.92 [0.90-0.94]	0.88 [0.86-0.91]	0.77 [0.73-0.81]	0.62 [0.53-0.71]
pDS vs. dDS	0.73 [0.68-0.78]	0.76 [0.71-0.80]	0.63 [0.58-0.69]	0.64 [0.58-0.69]	0.59 [0.52-0.67]
**Aβ status**					
Aβ+ vs. Aβ	0.95 [0.93-0.98]	0.95 [0.93-0.98]	0.90 [0.86-0.94]	0.76 [0.66-0.85]	0.58 [0.30-0.86]

AUC from ROC analyses discriminating clinical groups and CSF/ PET amyloid positive/ negative individuals. AUC and 95% CI are presented.

P-tau217 analyses were adjusted for age and sex using binary logistic regression models. AUCs are presented for prognostic value of age alone considering all ages, age >40 years and age >50 years in the DS cohort. aDS vs. dDS, *n* = 1154; aDS vs. pDS, *n* = 955; pDS vs. dDS, *n* = 483; Aβ+ vs. Aβ, *n* = 266.

Aβ, amyloid beta; aDS, asymptomatic Down syndrome; AUC, area under the curve; CI, confidence interval; CSF, cerebrospinal fluid; dDS, Down syndrome with dementia; pDS, presymptomatic Down syndrome; ROC, receiver operating characteristics

Supplementary Table S3. Accuracy of blood biomarkers in predicting clinical classification groups and amyloid status in DS and euploid individuals.

	DOWN SYNDROME COHORT		EUPLOID COHORT	
	**Clinical diagnosis**			
	AUC [95% CI]	Participant samples (N)	AUC [95% CI]	Participant samples (N)
**Biomarker**	**aDS vs. dDS**		**CN vs. AD**	
P-tau181	0.91 [0.89-0.93]	1020	0.85 [0.80-0.89]	345
P-tau231	0.87 [0.85-0.90]	1006	0.84 [0.80-0.89]	321
GFAP	0.95 [0.93-0.96]	914	0.92 [0.88-0.96]	316
NfL	0.93 [0.91-0.95]	867	0.86 [0.83-0.89]	517
	**aDS v. pDS**		**CN vs. MCI-AD**	
P-tau181	0.81 [0.77-0.86]	860	0.82 [0.77-0.87]	318
P-tau231	0.79 [0.74-0.83]	851	0.80 [0.75-0.85]	345
GFAP	0.83 [0.78-0.87]	777	0.86 [0.81-0.92]	290
NfL	0.83 [0.79-0.87]	734	0.77 [0.73-0.82]	464
	**pDS vs. dDS**		**MCI-AD vs. AD**	
P-tau181	0.69 [0.63-0.74]	420	0.55 [0.46-0.64]	173
P-tau231	0.67 [0.62-0.72]	413	0.60 [0.51-0.68]	161
GFAP	0.74 [0.69-0.80]	365	0.63 [0.54-0.71]	160
NfL	0.73 [0.67-0.78]	349	0.65 [0.59-0.71]	305
	**Amyloid status**			
	**Aβ+ vs. Aβ**		**Aβ+ vs. Aβ**	
P-tau181	0.81 [0.76-0.87]	278	0.83 [0.78-0.88]	323
P-tau231	0.79 [0.74-0.85]	264	0.82 [0.77-0.87]	294
GFAP	0.88 [0.83-0.93]	231	0.87 [0.83-0.91]	291
NfL	0.85 [0.80-0.90]	254	0.80 [0.76-0.84]	512

Reported is the AUC under the ROC curve and the 95% CI for discriminating diagnostic groups and amyloid status in the DS and euploid cohorts and the respective number (N) of participant samples used in the respective analysis

Aβ-, amyloid negative; Aβ+, amyloid positive, Aβ, amyloid beta; AD, Alzheimer's disease; aDS, asymptomatic Down syndrome; AUC, area under the curve of the ROC curve; CI, confidence interval; CN, cognitively normal; CSF, cerebrospinal fluid; dDS, Down syndrome with dementia; GFAP, glial fibrillary acidic protein; MCI, mild cognitive impairment; NfL, neurofilament light chain; pDS, presymptomatic Down syndrome; p-tau, phosphorylated tau; ROC, receiving operator characteristics

Supplementary Table S4. Accuracy of plasma p-tau217 vs. other blood biomarkers in predicting clinical diagnostic groups and amyloid status in individuals with Down syndrome and euploid individuals.

	p-tau217 vs. p-tau181		p-tau217 vs. p-tau231		p-tau217 vs. NfL		p-tau217 vs. GFAP	
	Difference between areas (mean [95% CI])	P-value*	Difference between areas (mean [95% CI])	P-value*	Difference between areas (mean [95% CI])	P-value*	Difference between areas (mean [95% CI])	P-value*
**Clinical diagnosis**								
aDS vs. dDS	0.05 [0.03-0.06]	<0.0001	0.09 [0.06-0.11]	<0.0001	0.03 [0.02-0.05]	<0.0001	0.02 [0.00-0.03]	0.0136
CN vs. AD	0.12 [0.08-0.15]	<0.0001	0.14 [0.09-0.18]	<0.0001	0.11 [0.08-0.15]	<0.0001	0.08 [0.04-0.11]	<0.0001
**Aβ status**								
DS Aβ+ vs. Aβ-	0.13 [0.08-0.17]	<0.0001	0.13 [0.08-0.18]	<0.0001	0.13 [0.06-0.16]	<0.0001	0.09 [0.04-0.14]	0.0003
Euploids Aβ+ vs. Aβ-	0.12 [0.07-0.17]	<0.0001	0.17 [0.12-0.23]	<0.0001	0.09 [0.05-0.14]	0.0001	0.07 [0.03-0.11]	0.0012

*AUC of ROC curves of demented vs. cognitively normal and amyloid positive vs. negative individuals in the DS and euploid cohort were compared using DeLong comparison (DeLong et al. 1988)

Aβ, amyloid beta; AD, Alzheimer's disease; aDS, asymptomatic Down syndrome; AUC, area under the curve; CI, confidence interval; CN, cognitively normal; dDS, Down syndrome with dementia; DS, Down syndrome; GFAP, glial fibrillary acidic protein; NfL, neurofilament light chain; p-tau, phosphorylated tau; ROC, receiving operator characteristics

Supplementary Table S5. Accuracy of combined blood biomarkers in predicting clinical classification groups and amyloid status in individuals with DS.

	p-tau217 + GFAP + NfL + p-tau181 + p-tau231		p-tau217 + GFAP + NfL		p-tau217 + GFAP		p-tau217 + NfL	
	**AUC [95% CI]**	**N**	**AUC [95% CI]**	**N**	**AUC [95% CI]**	**N**	**AUC [95% CI]**	**N**
**Clinical diagnosis**								
aDS vs. dDS	0.97 [0.96–0.98]	738	0.97 [0.96–0.98]	739	0.97 [0.96–0.98]	897	0.97 [0.96–0.98]	828
aDS v. pDS	0.91 [0.89–0.94]	629	0. 91 [0.88–0.94]	630	0.89 [0.85–0.92]	759	0.91 [0.89–0.94]	701
pDS vs. dDS	0.78 [0.73–0.84]	291	0.78 [0.73–0.83]	291	0.77 [0.72–0.82]	604	0.77 [0.71–0.82]	337
**Aβ status**								
Aβ+ vs. Aβ	0.95 [0.92-0.98]	198	0.95 [0.92-0.98]	198	0.95 [0.92-0.98]	224	0.95 [0.92-0.99]	228

Reported is the AUC under the ROC curve and the 95% CI for discriminating diagnostic groups and amyloid status in the DS and the respective number (N) of participant samples used in the respective analysis

The discriminative accuracy of biomarker combinations were evaluated using binary regression models and ROC analysis

Aβ-, amyloid negative; Aβ+, amyloid positive, Aβ, amyloid beta; AD, Alzheimer's disease; aDS, asymptomatic Down syndrome; AUC, area under the curve of the ROC curve; CI, confidence interval; CN, cognitively normal; CSF, cerebrospinal fluid; dDS, Down syndrome with dementia; GFAP, glial fibrillary acidic protein; MCI, mild cognitive impairment; NfL, neurofilament light chain; pDS, presymptomatic Down syndrome; p-tau, phosphorylated tau; ROC, receiving operator characteristic

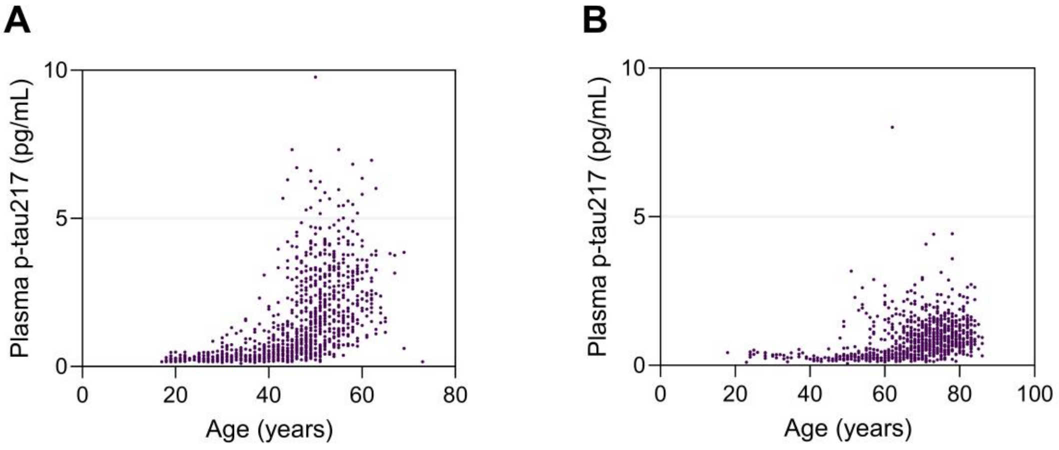

Supplementary Figure S1. Plasma p-tau217 levels across age.

Presented is a scatter plot of individual plasma-p-tau217 concentrations across age in individuals with (A) DS (*n* = 1291) and (B) euploid individuals (*n* = 963). Source data are provided as a Source Data file

DS, Down syndrome; p-tau217, phosphorylated tau

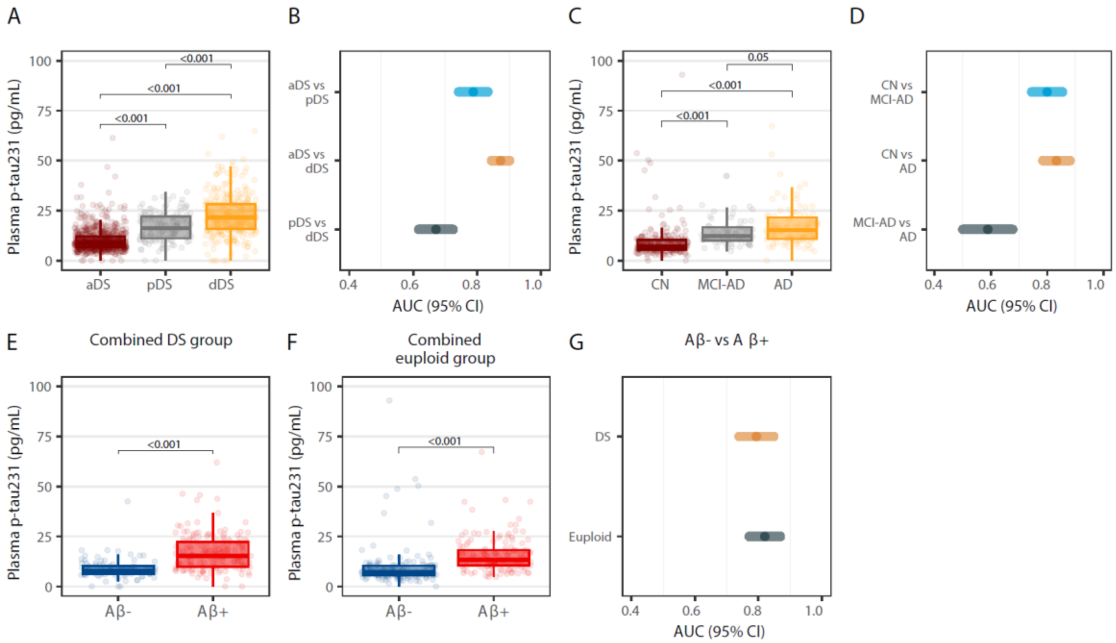

Supplementary Figure S2. Plasma levels and diagnostic accuracy of p-tau231 in individuals with DS and euploid controls with and without AD pathology.

Presented are the plasma concentrations (A+C) and the diagnostic performance (B+D) of p-tau231 in the diagnostic groups in individuals with Down syndrome and euploid controls. E and F show the plasma concentrations of p-tau231 in Aβ positive and negative individuals with (E) and without DS (F). In G, the diagnostic accuracy of plasma p-tau231 in discriminating Aβ positive and negative individuals in both cohorts is presented.

In panels A and C, the cognitively stable individuals are presented in burgundy, mildly cognitively impaired individuals in grey and individuals with dementia in yellow. In panels B, D and G, the cognitively stable group is plotted in cyan, the mildly cognitively impaired group in light peach and the dementia group in grey. In panels E and F, the Aβ- group is displayed in blue, the Aβ+ group is displayed in red. aDS *n* = 722, pDS *n* = 129, dDS *n* = 284, CN *n* = 227, MCI-AD *n* = 67, AD *n* = 94, DS Aβ+ *n* = 172, DS Aβ- *n* = 92, euploid Aβ+ *n* = 141, euploid Aβ- *n* = 153. Exact p-values: Panel A: aDS vs pDS, p = 3.65E-18; aDS vs dDS, p = 2.37E-54; pDS vs dDS, p = 3.41E-10; panel C: CN vs MCI, p = 4.26E-06; CN vs AD, p = 5.57E-10; MCI vs AD, p = 0.04509; panel E: p = 1.33E-15, panel F: p = 8.45E-08.

P-values were derived from two-sided independent t-tests for pairwise group comparisons; p-values were not corrected for multiple testing. Boxplots display the median, IQR (bounds of the box), and whiskers extending to the minimum and maximum values within 1.5 × IQR; individual data points are shown with jittered dots (A, C, E, F). The performance of plasma biomarkers in predicting the diagnostic and Aβ groups was analyzed using AUC ROC analyses; the AUC and respective 95% CI are presented in the forest plot (B, D, G). Source data are provided as a Source Data file.

Aβ, amyloid beta; AD, Alzheimer's disease; aDS, asymptomatic Down syndrome; AUC, area under the curve; CI, confidence interval; CN, cognitively normal; CSF, cerebrospinal fluid; dDS, Down syndrome with dementia; MCI, mild cognitive impairment; pDS, presymptomatic Down syndrome; p-tau, phosphorylated tau; ROC, receiver operating characteristics

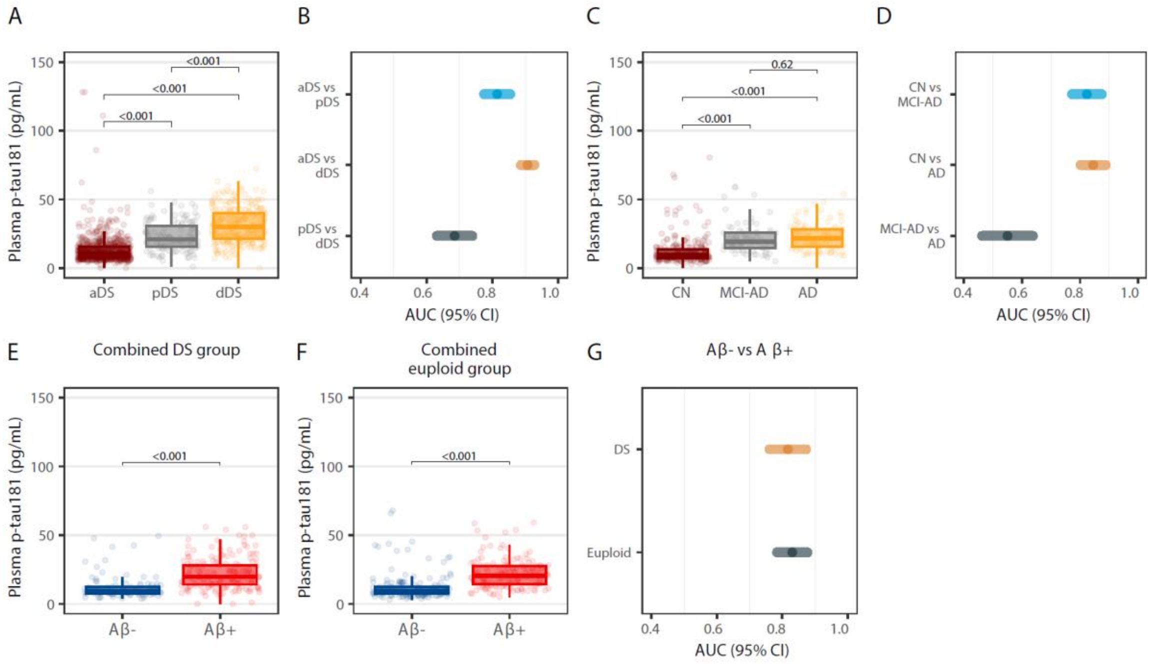

Supplementary Figure S3. Plasma levels and diagnostic accuracy of p-tau181 in individuals with DS and euploid controls with and without AD pathology.

Presented are the plasma concentrations (A+C) and the diagnostic performance (B+D) of p-tau181 in the diagnostic groups in individuals with Down syndrome and euploid controls. E and F show the plasma concentrations of p-tau181 in Aβ positive and negative individuals with (E) and without DS (F). In G, the diagnostic accuracy of plasma p-tau181 in discriminating Aβ positive and negative individuals in both cohorts is presented

In panels A and C, the cognitively stable individuals are presented in burgundy, mildly cognitively impaired individuals in grey and individuals with dementia in yellow. In panels B, D and G, the cognitively stable group is plotted in cyan, the mildly cognitively impaired group in light peach and the dementia group in grey. In panels E and F, the Aβ- group is displayed in blue, the Aβ+ group is displayed in red. aDS *n* = 730, pDS *n* = 130, dDS *n* = 290, CN *n* = 245, MCI-AD *n* = 73, AD *n* = 100, DS Aβ+ *n* = 179, DS Aβ- *n* = 99, euploid Aβ+ *n* = 151, euploid Aβ- *n* = 172. Exact p-values: Panel A: aDS vs pDS, p = 5.73E-19; aDS vs dDS, p = 1.78E-71; pDS vs dDS, p = 1.23E-10; panel C: CN vs MCI, p = 3.63E-09; CN vs AD, p = 1.43E-15; MCI vs AD, p = 0.617073; panel E: p = 2.60E-14, panel F: p = 6.05E-16.

P-values were derived from two-sided independent t-tests for pairwise group comparisons; p-values were not corrected for multiple testing. Boxplots display the median, IQR (bounds of the box), and whiskers extending to the minimum and maximum values within 1.5 × IQR; individual data points are shown with jittered dots (A, C, E, F). The performance of plasma biomarkers in predicting the diagnostic and Aβ groups was analyzed using AUC ROC analyses; the AUC and respective 95% CI are presented in the forest plot (B, D, G). Source data are provided as a Source Data file

Aβ, amyloid beta; AD, Alzheimer's disease; aDS, asymptomatic Down syndrome; AUC, area under the curve; CI, confidence interval; CN, cognitively normal; CSF, cerebrospinal fluid; dDS, Down syndrome with dementia; MCI, mild cognitive impairment; pDS, presymptomatic Down syndrome; p-tau, phosphorylated tau; ROC, receiver operating characteristics

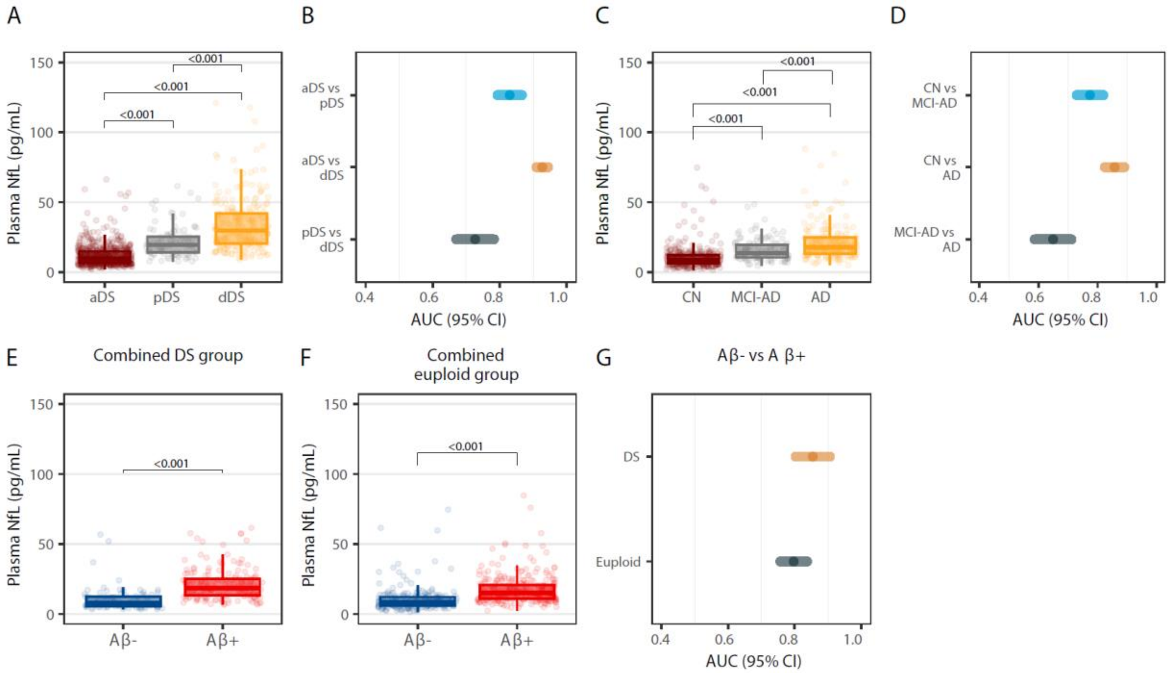

Supplementary Figure S4. Plasma levels and diagnostic accuracy of NfL in individuals with DS and euploid controls with and without AD pathology.

Presented are the plasma concentrations (A+C) and the diagnostic performance (B+D) of NfL in the diagnostic groups in individuals with Down syndrome and euploid controls. E and F show the plasma concentrations of NfL in Aβ positive and negative individuals with (E) and without DS (F). In G, the diagnostic accuracy of plasma NfL in discriminating Aβ positive and negative individuals in both cohorts is presented

In panels A and C, the cognitively stable individuals are presented in burgundy, mildly cognitively impaired individuals in grey and individuals with dementia in yellow. In panels B, D and G, the cognitively stable group is plotted in cyan, the mildly cognitively impaired group in light peach and the dementia group in grey. In panels E and F, the Aβ- group is displayed in blue, the Aβ+ group is displayed in red. aDS n = 626, pDS n = 108, dDS n = 241, CN n = 338, MCI-AD n = 126, AD n = 179, DS Aβ+ n = 166, DS Aβ- n = 88, euploid Aβ+ n = 291, euploid Aβ- n = 221. Exact p-values: Panel A: aDS vs pDS, p = 5.28E-16; aDS vs dDS, p = 1.16E-43; pDS vs dDS, p = 6.06E-13; panel C: CN vs MCI, p = 1.30E-10; CN vs AD, p = 6.14E-10; MCI vs AD, p = 0.000444; panel E: p = 3.22E-15, panel F: p = 1.73E-09.

P-values were derived from two-sided independent t-tests for pairwise group comparisons; p-values were not corrected for multiple testing. Boxplots display the median, IQR (bounds of the box), and whiskers extending to the minimum and maximum values within 1.5 × IQR; individual data points are shown with jittered dots (A, C, E, F). The performance of plasma biomarkers in predicting the diagnostic and Aβ groups was analyzed using AUC ROC analyses; the AUC and respective 95% CI are presented in the forest plot (B, D, G). Source data are provided as a Source Data file

Aβ, amyloid beta; AD, Alzheimer's disease; aDS, asymptomatic Down syndrome; AUC, area under the curve; CI, confidence interval; CN, cognitively normal; CSF, cerebrospinal fluid; dDS, Down syndrome with dementia; MCI, mild cognitive impairment; NfL, neurofilament light; pDS, presymptomatic Down syndrome; ROC, receiver operating characteristics

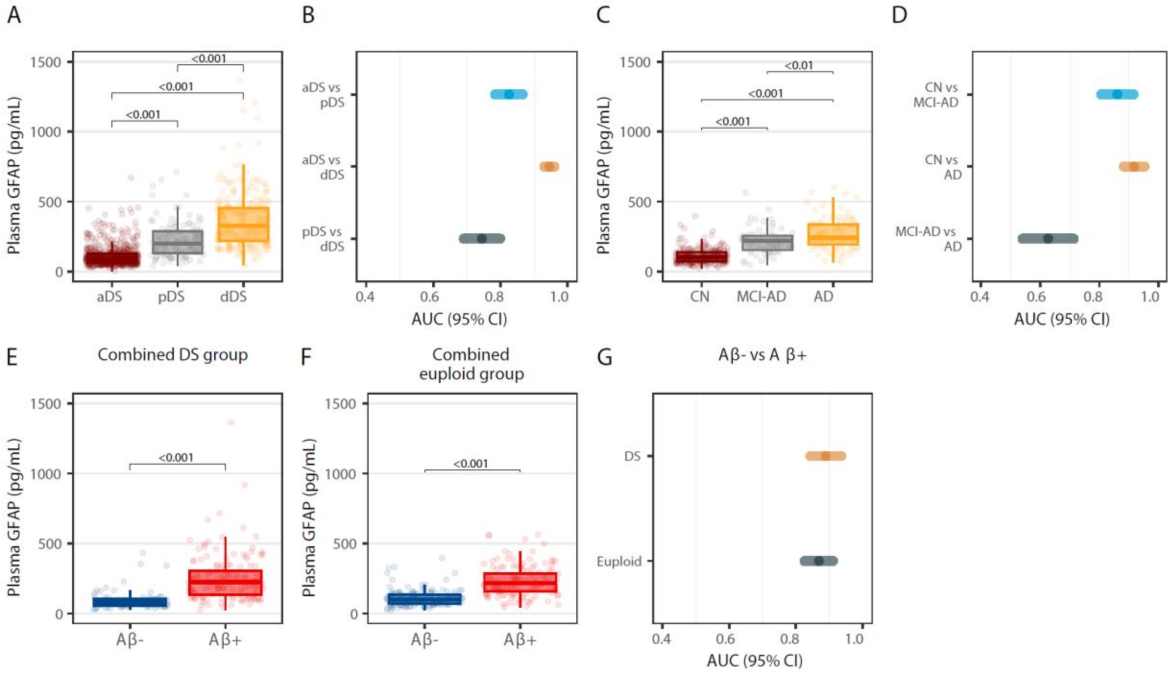

Supplementary Figure S5. Plasma levels and diagnostic accuracy of GFAP in individuals with DS and euploid controls with and without AD pathology.

Presented are the plasma concentrations (A+C) and the diagnostic performance (B+D) of GFAP in the diagnostic groups in individuals with Down syndrome and euploid controls. E and F show the plasma concentrations of GFAP in Aβ positive and negative individuals with (E) and without DS (F). In G, the diagnostic accuracy of plasma GFAP in discriminating Aβ positive and negative individuals in both cohorts is presented

In panels A and C, the cognitively stable individuals are presented in burgundy, mildly cognitively impaired individuals in grey and individuals with dementia in yellow. In panels B, D and G, the cognitively stable group is plotted in cyan, the mildly cognitively impaired group in light peach and the dementia group in grey. In panels E and F, the Aβ- group is displayed in blue, the Aβ+ group is displayed in red. aDS *n* = 663, pDS *n* = 114, dDS *n* = 251, CN *n* = 223, MCI-AD *n* = 67, AD *n* = 93, DS Aβ+ *n* = 147, DS Aβ- *n* = 84, euploid Aβ+ *n* = 140, euploid Aβ- *n* = 151. Exact p-values: Panel A: aDS vs pDS, p = 3.77E-16; aDS vs dDS, p = 4.76E-53; pDS vs dDS, p = 1.14E-14; panel C: CN vs MCI, p = 9.22E-14; CN vs AD, p = 1.96E-23; MCI vs AD, p = 0.002393; panel E: p = 1.21E-19, panel F: p = 9.99E-26.

P-values were derived from two-sided independent t-tests for pairwise group comparisons; p-values were not corrected for multiple testing. Boxplots display the median, IQR (bounds of the box), and whiskers extending to the minimum and maximum values within 1.5 × IQR; individual data points are shown with jittered dots (A, C, E, F). The performance of plasma biomarkers in predicting the diagnostic and Aβ groups was analyzed using AUC ROC analyses; the AUC and respective 95% CI are presented in the forest plot (B, D, G). Source data are provided as a Source Data file

Aβ, amyloid beta; AD, Alzheimer's disease; aDS, asymptomatic Down syndrome; AUC, area under the curve; CI, confidence interval; CN, cognitively normal; CSF, cerebrospinal fluid; dDS, Down syndrome with dementia; GFAP, glial fibrillary acidic protein; MCI, mild cognitive impairment; NfL, neurofilament light; pDS, presymptomatic Down syndrome; ROC, receiver operating characteristics
