# Supplementary material for: Plasma p-tau217 as a biomarker of Alzheimer’s disease pathology in individuals with Down syndrome
Source: Nat Commun. 2025 Nov 10;16:9900. doi: 10.1038/s41467-025-65882-x (PMC12603275; doi:10.1038/s41467-025-65882-x)
Supplement: Supplementary file 2 — Reporting Summary [file 41467_2025_65882_MOESM2_ESM.pdf]

nature portfolio

Corresponding author(s): N. J. Ashton

Last updated by author(s): Oct 18, 2025

Reporting Summary

Nature Portfolio wishes to improve the reproducibility of the work that we publish. This form provides structure for consistency and transparency in reporting. For further information on Nature Portfolio policies, see our Editorial Policies and the Editorial Policy Checklist.

Statistics

For all statistical analyses, confirm that the following items are present in the figure legend, table legend, main text, or Methods section.

n/a	Confirmed	
☐	☒	The exact sample size (*n*) for each experimental group/condition, given as a discrete number and unit of measurement
☐	☒	A statement on whether measurements were taken from distinct samples or whether the same sample was measured repeatedly
☐	☒	The statistical test(s) used AND whether they are one- or two-sided<br>*Only common tests should be described solely by name; describe more complex techniques in the Methods section.*
☐	☒	A description of all covariates tested
☐	☒	A description of any assumptions or corrections, such as tests of normality and adjustment for multiple comparisons
☐	☒	A full description of the statistical parameters including central tendency (e.g. means) or other basic estimates (e.g. regression coefficient) AND variation (e.g. standard deviation) or associated estimates of uncertainty (e.g. confidence intervals)
☐	☒	For null hypothesis testing, the test statistic (e.g. *F*, *t*, *r*) with confidence intervals, effect sizes, degrees of freedom and *P* value noted<br>*Give P values as exact values whenever suitable.*
☒	☐	For Bayesian analysis, information on the choice of priors and Markov chain Monte Carlo settings
☒	☐	For hierarchical and complex designs, identification of the appropriate level for tests and full reporting of outcomes
☐	☒	Estimates of effect sizes (e.g. Cohen's *d*, Pearson's *r*), indicating how they were calculated

Our web collection on statistics for biologists contains articles on many of the points above.

Software and code

Policy information about availability of computer code

Data collection	No software was used
Data analysis	GraphPad Prism 9 for Windows, MedCalc version 23.0.9, and R version 4.3.3. The following R packages have been used: ggplot2, dplyr, ggsignif, pROC, ggsci

For manuscripts utilizing custom algorithms or software that are central to the research but not yet described in published literature, software must be made available to editors and reviewers. We strongly encourage code deposition in a community repository (e.g. GitHub). See the Nature Portfolio guidelines for submitting code & software for further information.

Data

Policy information about availability of data

All manuscripts must include a data availability statement. This statement should provide the following information, where applicable:

- Accession codes, unique identifiers, or web links for publicly available datasets
- A description of any restrictions on data availability
- For clinical datasets or third party data, please ensure that the statement adheres to our policy

Due to consenting issues and IRB requirements, we cannot make the dataset publicly available in a repository, as explicit consent for public sharing was only introduced recently in our cohort and is therefore not in place for many participants. However, the DABNI cohort has supported dozens of international collaborations, and data can be shared following standard data transfer agreements. Requests can be directed to Juan Fortea (jfortea@santpau.cat) or Ana Bueno

(abueno@santpau.cat). Access is limited to bona fide researchers for scientific purposes, and requests are typically processed and accepted within 2-3 months. Biomarker data displayed in this study are provided in the Supplementary Information/Source Data file.

Research involving human participants, their data, or biological material

Policy information about studies with human participants or human data. See also policy information about sex, gender (identity/presentation), and sexual orientation and race, ethnicity and racism.

Reporting on sex and gender

Biological sex was determined based on self-identification at the first visit; the term sex (biological attribute) was used in the manuscript.

Reporting on race, ethnicity, or other socially relevant groupings

NA

Population characteristics

Adults with Down syndrome (DS) and euploid controls aged 18 years and older with and without symptomatic Alzheimer's disease (AD) were included.
DS cohort: In a systematic and structured process, individuals with DS were independently classified by two blinded neurologists and neuropsychologists (without access to AD biomarker results but with access to basic neuroimaging and blood analytics) in one of three diagnostic categories: 1) Asymptomatic or cognitively stable (no clinical or neuropsychological suspicion of symptomatic AD); 2) Prodromal AD (suspicion of cognitive deterioration due to AD, but symptoms do not fulfill the criteria for dementia) ; 3) AD dementia (AD dementia with cognitive decline and impaired activities of daily living including worsening from baseline level of independence). This systematic approach ensures robust diagnostic validity while minimizing potential biases.
Euploid cohort: Participants were classified as cognitively normal controls (no memory complaints, MMSE 27–30, CDR global score = 0, FCSRT total immediate score (EAS62) ≥ 7, absence of significant impairment in other domains or in daily living activities), prodromal dementia (MMSE 24–30, CDR global score = 0.5, absence of a clinical diagnosis of dementia, CSF biomarkers supporting AD pathophysiology) or AD dementia (CDR global score ≥ 0.5, FCSRT total immediate score (EAS62) ≤ 6, clinical criteria of “probable AD dementia with evidence of the AD pathophysiological process”)

Recruitment

For this cross-sectional study, adults with DS and euploid controls in Barcelona were recruited at the Hospital of Sant Pau, Barcelona, Spain. We included all individuals who had at least one blood-based AD biomarker and were classified as asymptomatic/ cognitively stable, mild cognitive impairment or AD dementia in the present study.

Down Syndrome cohort: Individuals with Down syndrome aged 18 years or older were recruited from a population-based health plan designed to screen for AD dementia, which includes yearly neurological and neuropsychological assessments. Individuals in this plan interested in research studies are included in the Down Alzheimer Barcelona Neuroimaging Initiative (DABNI) cohort (Fortea et al. 2020, PMID: 32593336). The Alzheimer Down Unit is the reference unit in Catalonia for neurological pathology associated with DS. Around 3500 adults with DS are estimated to live in Catalonia. To date, the Alzheimer-Down Unit has evaluated more than 1100 individuals with DS. The health plan underpinning the DABNI initiative, developed for adults with DS has been disseminated in different foundations, residences, occupational centers, and special employment centers to ensure a more representative population-based sample. Moreover, for patients who are unable to physically attend the study center, the Domiciliary Alzheimer Visiting in Down Syndrome (DAVIS) program was developed, which allows to reach different centers in Catalonia to evaluate individuals with difficulties in traveling to Barcelona, seeking to provide access to medical, neurological, and neuropsychological assessments.

Euploid cohort: Non-trisomic individuals aged 18 years or older up to 75 years from the Sant Pau Initiative on Neurodegeneration (SPIN). The Sant Pau Memory Unit attends patients with cognitive or behavioral symptoms referred either by their primary care physician or by other neurologists to receive specialized diagnosis and treatment and/or to facilitate their participation in research protocols. Euploid controls underwent a structured neurological assessment and a comprehensive battery of neuropsychological tests to establish normal cognitive health. The SPIN cohort used in this study includes cognitively normal participants (no memory complaints, MMSE 27-30, CDR global=0, EAS62 >=7, no impairments in daily living activities), mild cognitive impairment (MMSE 24-30, CDS global =0.5, no dementia diagnosis, CSF AD biomarker positive) and AD dementia (CDR >= 0.5, EAS62 <= 6) (Alcolea et al. 2019, PMID: 31650016).
The exclusion criteria were: Inability to complete neuropsychological tests and questionnaires (illiteracy, blindness, hearing impairment, contraindication for MRI (claustrophobia, pacemaker, aneurism clips, cardiac mechanical valve), contraindication for lumbar puncture (anticoagulation, coagulation disease), anticoagulant treatment, current treatment with drugs that can impair cognition, medical history of neurological disease (stroke, brain lesions, epilepsy), psychiatric disease (psychosis or major depression), drug abuse in the last year, medical history of cancer (if affecting the central nervous system, it has not been in complete remission for >=5 years, potentially neurotoxic chemotherapy or cranial radio therapy was received).

Participants were recruited following referral by primary care physicians or neurologists/ a population-based health plan, which may introduce recruitment bias toward individuals with greater healthcare access, higher health awareness, or more advanced or recognized symptoms.

Ethics oversight

Sant Pau Ethical Review Authority (IBSP-NGF-2018-36 and IIBSP-DOW-2014-30)

Note that full information on the approval of the study protocol must also be provided in the manuscript.

Field-specific reporting

Please select the one below that is the best fit for your research. If you are not sure, read the appropriate sections before making your selection.

☒ Life sciences ☐ Behavioural & social sciences ☐ Ecological, evolutionary & environmental sciences

For a reference copy of the document with all sections, see nature.com/documents/nr-reporting-summary-flat.pdf

Life sciences study design

All studies must disclose on these points even when the disclosure is negative.

Sample size	Included were all individuals from the Down Alzheimer Barcelona Neuroimaging Initiative (DABNI) and Sant Pau Initiative on Neurodegeneration (SPIN) with AD-related blood biomarker data available. No samples size calculation was performed.
Data exclusions	Participants who were categorized as "uncertain" category during the diagnostic process (cognitive or daily living impairments are attributed to medical, pharmacological, or psychiatric conditions rather than a neurodegenerative origin) were excluded from this study. No data was excluded during data analysis.
Replication	This study was designed as a large and extensively characterized observational analysis (n=2329). Results of the plasma analysis were not replicated due to the unique nature of the DS cohort and plasma availability.
Randomization	This is an observational study and no allocation into experimental groups has been performed. Therefore, randomization is not relevant for this study.
Blinding	Investigators were blinded to diagnosis during plasma processing and data acquisition. Data analysis was necessarily performed unblinded to diagnosis on de-identified data.

Reporting for specific materials, systems and methods

We require information from authors about some types of materials, experimental systems and methods used in many studies. Here, indicate whether each material, system or method listed is relevant to your study. If you are not sure if a list item applies to your research, read the appropriate section before selecting a response.

Materials & experimental systems

n/a	Involved in the study	
☐	☒	Antibodies
☒	☐	Eukaryotic cell lines
☒	☐	Palaeontology and archaeology
☒	☐	Animals and other organisms
☒	☐	Clinical data
☒	☐	Dual use research of concern
☒	☐	Plants

Methods

n/a	Involved in the study	
☒	☐	ChIP-seq
☒	☐	Flow cytometry
☒	☐	MRI-based neuroimaging

Antibodies

Antibodies used	Plasma NfL and GFAP were measured by the commercial Neurology 2-Plex B kit for Simoa HD-X as described in Ashton et al. Nat Commun 2021 (PMID: 34099648) and Montoliu-Gaya et al. EBioMedicine 2023 (PMID: 37002988). Plasma pTau231 and plasma p-tau181 was measured by two in-house Simoa assays developed at the University of Gothenburg as described in Ashton et al. Acta Neuropathol 2021 (PMID: 33585983) and Karikari et al. Lancet Neurol 2020 (PMID: 32333900). Plasma p-tau217 was measured with the commercial ALZpath pTau217 assay for Simoa HD-X as described in Ashton et al, JAMA Neurol 2024 (PMID: 38252443). CSF measures of Abeta1-40 and Abeta1-42 were obtained on the automated Lumipulse G600II platform (Fujirebio) as previously described in Fortea et al., Lancet Neurol 2018 (PMID: 32593336).
Validation	Please see aforementioned studies for further details.

Plants

Seed stocks	*Report on the source of all seed stocks or other plant material used. If applicable, state the seed stock centre and catalogue number. If plant specimens were collected from the field, describe the collection location, date and sampling procedures.*
Novel plant genotypes	*Describe the methods by which all novel plant genotypes were produced. This includes those generated by transgenic approaches, gene editing, chemical/radiation-based mutagenesis and hybridization. For transgenic lines, describe the transformation method, the number of independent lines analyzed and the generation upon which experiments were performed. For gene-edited lines, describe the editor used, the endogenous sequence targeted for editing, the targeting guide RNA sequence (if applicable) and how the editor was applied.*
Authentication	*Describe any authentication procedures for each seed stock used or novel genotype generated. Describe any experiments used to assess the effect of a mutation and, where applicable, how potential secondary effects (e.g. second site T-DNA insertions, mosiacism, off-target gene editing) were examined.*
