# Supplementary material for: Plasma p-tau217 as a biomarker of Alzheimer’s disease pathology in individuals with Down syndrome
Source: Nat Commun. 2025 Nov 10;16:9900. doi: 10.1038/s41467-025-65882-x (PMC12603275; doi:10.1038/s41467-025-65882-x)
Supplement: Supplementary file 3 — Transparent Peer Review File [file 41467_2025_65882_MOESM3_ESM.pdf]

nature portfolio

Peer Review File

Plasma pTau217 as a Biomarker of Alzheimer’s Disease Pathology in Individuals with Down Syndrome

Corresponding Author: Dr Nicholas Ashton

This manuscript has been previously reviewed at another journal. This document only contains information relating to versions considered at Nature Communications.

This file contains all reviewer reports in order by version, followed by all author rebuttals in order by version.

Version 0:

Reviewer comments:

Reviewer #1

(Remarks to the Author)
The authors have satisfactorily responded to all of the reviewer comments and suggestions. The paper is now acceptable for publication.

Open Access This Peer Review File is licensed under a Creative Commons Attribution 4.0 International License, which permits use, sharing, adaptation, distribution and reproduction in any medium or format, as long as you give appropriate credit to the original author(s) and the source, provide a link to the Creative Commons license, and indicate if changes were made.
In cases where reviewers are anonymous, credit should be given to 'Anonymous Referee' and the source.
The images or other third party material in this Peer Review File are included in the article’s Creative Commons license, unless indicated otherwise in a credit line to the material. If material is not included in the article’s Creative Commons license and your intended use is not permitted by statutory regulation or exceeds the permitted use, you will need to obtain

permission directly from the copyright holder.
To view a copy of this license, visit https://creativecommons.org/licenses/by/4.0/

REVIEWERS' COMMENTS

Reviewer #1 (Remarks to the Author):

The authors have satisfactorily responded to all of the reviewer comments and suggestions. The paper is now acceptable for publication.

> We thank Reviewer one for the positive evaluation and the time and effort invested in the review process.
